# Highly photoresponsive and wavelength-selective circularly-polarized-light detector based on metal-oxides hetero-chiral thin film

**DOI:** 10.1038/srep19580

**Published:** 2016-01-22

**Authors:** Seung Hee Lee, Dhruv Pratap Singh, Ji Ho Sung, Moon-Ho Jo, Ki Chang Kwon, Soo Young Kim, Ho Won Jang, Jong Kyu Kim

**Affiliations:** 1 Department of Materials Science and Engineering, Pohang University of Science and Technology (POSTECH), Pohang, Gyeongbuk, 790-784, Republic of Korea; 2 Max Planck Institute for Intelligent Systems, Heisenbergstrasse 3, 70569 Stuttgart, Germany; 3 School of Chemical Engineering and Materials Science, Chung-Ang University, Seoul, 156-756, Republic of Korea; 4 Department of Materials Science and Engineering, Research Institute for Advanced Materials, Seoul National University, Seoul, 151-744, Republic of Korea

## Abstract

A highly efficient circularly-polarized-light detector with excellent wavelength selectivity is demonstrated with an elegant and simple microelectronics-compatible way. The circularly-polarized-light detector based on a proper combination of the geometry-controlled TiO_2_-SnO_2_ hetero-chiral thin film as an effective chiroptical filter and the Si active layer shows excellent chiroptical response with external quantum efficiency as high as 30% and high helicity selectivity of ~15.8% in an intended wavelength range. Furthermore, we demonstrated the ability of manipulating both bandwidth and responsivity of the detector simultaneously in whole visible wavelength range by a precise control over the geometry and materials constituting hetero-chiral thin film. The high efficiency, wavelength selectivity and compatibility with conventional microelectronics processes enabled by the proposed device can result in remarkable developments in highly integrated photonic platforms utilizing chiroptical responses.

Over the past decades, circularly-polarized-light carrying photons with spin angular momentum has intrigued tremendous interests in both classical and quantum photonic technologies. The light-matter interaction between the helicity (left- or right-handedness) of the circularly-polarized-light and the spin states of the electrons leads to open up possibilities for a wide range of applications such as optical communication of spin information^1^,^2^,^3^,^4^, spin-state control in quantum information technologies^5^,^6^,^7^, and ellipsometric tomography^8^,^9^. In order to realize full potential of these technologies, there have been increasing demands on miniaturized devices incorporated into a highly integrated photonic platform by using conventional microelectronics processes to detect circularly-polarized-light with high quantum efficiency and selectivity in an intended wavelength range.

A helically shaped nanostructure can couple directly to circularly-polarized-light, thus show a chiral response based on difference in either absorbance or reflectance/transmittance for the handedness of circularly-polarized-light. Recently, organic field-effect transistors based on a thin film consisting of aligned chiral semiconducting molecules known as helicene have been demonstrated to show a specific photoresponse to circularly-polarized-light near the absorption wavelength band, which is directly related to the handedness of the helicene molecule^10^. However, since the chiroptical responding wavelength is determined by the intrinsic chemical structure of the chiral molecule, it would be difficult to tune the detection wavelength to an intended range. In addition, low quantum efficiency (~0.1%) even at high operating voltages over 10 V caused by relatively poor in-plane charge transport perpendicular to the direction of aligned chiral molecules, as well as difficulty in integration of such devices using conventional microelectronic techniques call for a new approach for the realization of a miniaturized device with high quantum efficiency, controllable detection wavelengths, and compatibility with conventional integrated circuit technologies.

Besides such an absorption-based chiral response, an array of metal-oxide nano helices can exhibit the circular Bragg phenomenon, i.e., the incident circularly-polarized-light will be reflected (or transmitted) if the handedness of the polarization is the same (or the opposite) as that of the helices. This effect appears due to the birefringent nature of chiral objects and occurs at the wavelengths under circular Bragg regime^11^,^12^,^13^. The central wavelength of the circular Bragg regime (*λ*_*0*_^*Bragg*^) is given by:


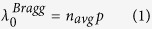


where *n*_*avg*_ is average refractive index of the film, *p* is pitch of the helices^13^,^14^. M. J. Brett and A. Lakhtakia research groups have theoretically designed and fabricated single-layer chiral thin films with successive pitches forming a geometric series by using GLAD method, and demonstrated that the *λ*_*0*_^*Bragg*^ of a single-layer chiral film can be tuned to a desired wavelength by controlling pitch and/or by choosing a material with an appropriate refractive index^13^,^15^,^16^,^17^. However, the chiroptical properties of hetero-chiral films consisting of multi-layered materials with different refractive indices which can be utilized to realize an integrated optical (circularly-polarized light)-to-electrical transducer with excellent wavelength tunability and broadband characteristics have not been reported. With a proper combination of conventional inorganic semiconductor active layer materials having an excellent carrier transport property and grown multi-layered hetero-chiral films in which the pitch and the refractive index of each layer can be precisely controlled on demand^18^,^19^,^20^,^21^, a high performance circularly-polarized-light detector active for an intended wavelength integrated on a photonic circuit can be realized.

In this paper, we present circularly-polarized-light detectors based on hetero-chiral films consisting of different refractive indices materials (titanium dioxide (TiO_2_) and tin dioxide (SnO_2_)) with precisely controlled geometry as chiroptical filters on silicon (Si) active layers. The detector shows external quantum efficiency as high as ~30%, and excellent selectivity in detection wavelengths, which are attributed to the Si photo-generating active layer with high carrier mobility and the geometry-controlled TiO_2_-SnO_2_ hetero-chiral film, respectively. We believe that it opens up possibilities for development of highly integrated photonic devices having excellent chiroptical response with high selectivity in an intended wavelength range with a simple microelectronics-compatible way.

## Results and Discussion

### Geometric properties

Glancing angle deposition (GLAD) method was used to grow the hetero-chiral thin films over Si substrates. GLAD is a well-established physical vapor deposition method to grow the chiral films of a wide range of materials. In this method, the pitch of chiral nano helices can be easily modified by controlling some growth parameters like, substrate-tilt angle, substrate-rotation speed, and deposition rate. Following the Equation (1), this control over the pitch (*p*) and average refractive index (*n*_*avg*_) provides a precise tuning of central wavelength of the circular Bragg regime (*λ*_*0*_^*Bragg*^) of a single-layer chiral thin film to a desired value^15^. Furthermore, various hetero-chiral films can be easily constructed by sequential GLAD of different materials with appropriate combination of refractive indices and geometric shapes, which can expand the degrees of freedom on the locations of *λ*_*0*_^*Bragg*^ as well as the bandwidth of the Bragg regime, thus can enhance the chiroptical response within a desired wavelength range. The chiroptical response of a chiral film normally increases with the number of helical turns and with increasing refractive index of the film^12^,^15^. However for some materials the GLAD suffers with the helix shape broadening and film porosity increment with the length or number of turns. This effect increases the diffuse scattering and hence dampens the chiroptical signal. In this study we used 5 turns helices and TiO_2_ and SnO_2_ are selected as high refractive index materials (*n*_*avg,TiO2*_ ~1.8 and *n*_*avg,SnO2*_ ~1.6 for chiral films fabricated by GLAD with substrate tilting angle of 65°).

**Figure 1:**
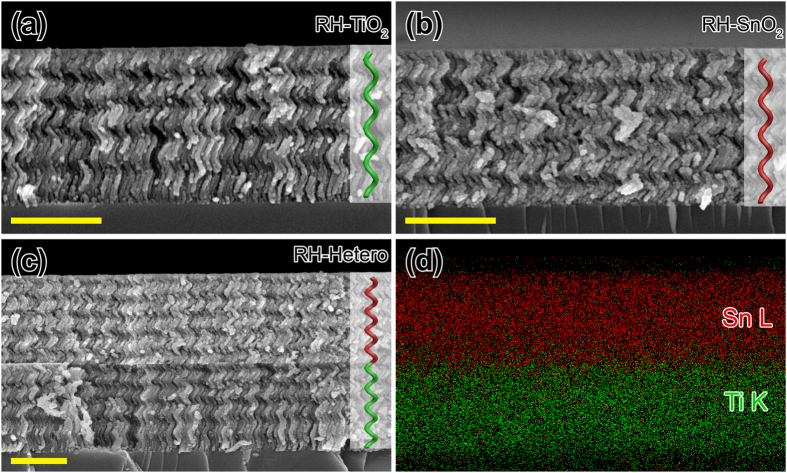
SEM images and EDS maps of metal-oxides chiral films. (**a–c**) Cross-sectional SEM images of right-handed (RH) TiO_2_, SnO_2_, and TiO_2_-SnO_2_ hetero-chiral films, respectively. Insets show corresponding schematic structures of the individual helix. All scale bars are 1 μm. (**d**) EDS maps of the hetero-chiral film shown in (**c**).

Figure 1a–c show cross-sectional scanning electron microscopic (SEM) images of the metal-oxides right-handed (RH) chiral films on Si wafers with corresponding schematics of a single helix. The *p* of 1.7 μm thick TiO_2_ (Fig. 1a) and SnO_2_ (Fig. 1b) chiral films composed of five turn helices are estimated to be 340 nm. The circular Bragg phenomenon from each sample is expected to occur at 612 and 544 nm according to Equation (1). Figure 1c shows cross-sectional SEM image of RH TiO_2_-SnO_2_ hetero-chiral film fabricated by sequential GLAD on a Si wafer. Two layers are clearly distinguished by the contrast caused by the difference in electron density between Ti (low electron density, dark) and Sn (high electron density, bright). Figure 1d shows energy-dispersive X-ray spectroscopy maps of the hetero-chiral film shown in Fig. 1c which clearly confirms the presence of Ti and Sn elements in each layer of the bilayer film. These results confirm the successful fabrication of TiO_2_-SnO_2_ hetero-chiral film by sequential GLAD.

### Chiroptical properties

**Figure 2:**
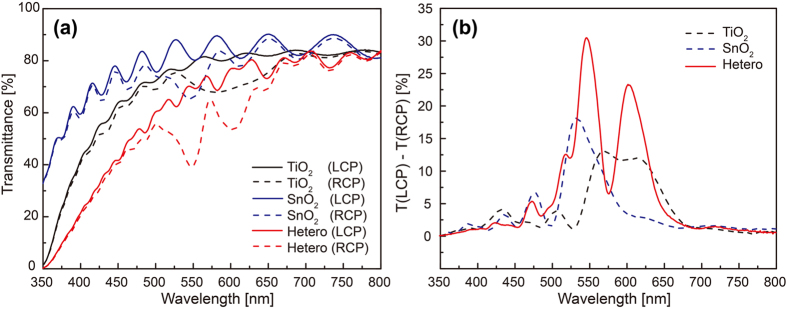
Transmittance spectra of circularly-polarized-light through metal-oxides chiral films and their differences. (**a**) Transmittance spectra of LCP (solid lines) and RCP (dotted lines) light through the TiO_2_ (black), the SnO_2_ (blue), and the hetero (red)-chiral films. (**b**) Difference in the transmitted LCP and RCP light through the chiral films indicating the magnitude of chiroptical responses. Chiroptical response of the hetero-chiral film (solid line) results from the combination of that of both single chiral films (dotted lines).

Figure 2a shows the transmittance spectra of right handed circularly-polarized (RCP, solid lines)- and left handed circularly-polarized (LCP, dotted lines)-light through the TiO_2_ and the SnO_2_ single-layer chiral films, and the TiO_2_-SnO_2_ hetero-chiral film on a glass substrate as a function of the wavelength of incident light. All the spectra show fringes due to the interference between the films and the glass substrate. Note that the hetero-chiral film shows irregular fringes and lowest transmittance among three films due to the interference at three interfaces and larger optical absorption and scattering loss, respectively. It can be noticed that RCP light meets with comparatively low transmittance at certain wavelengths for all the three chiral films. This is because the RH chiral films preferentially transmit incident circularly-polarized-light of opposite handedness (LCP light) while reflecting that of same handedness (RCP light) near the circular Bragg regime.

Figure 2b shows the difference in the transmittance of LCP and RCP light through the three chiral films estimated from Fig. 2a, representing the magnitude of the chiroptical response of each film. Peaks located at *λ*_*0*_^*Bragg*^ indicate the circular Bragg regimes where the circularly-polarized-light interacts predominantly with the birefringence nature of the chiral film. Despite the same *p* of the TiO_2_ and the SnO_2_ chiral films as shown in Fig. 1, the *λ*_*0*_^*Bragg*^’^s^ are different from each other due to the difference in the *n*_*avg*_ of each film, showing a possibility of selecting the detection wavelength of interest simply by choosing a proper material. The magnitude of chiroptical response of SnO_2_ chiral film having lower *n*_*avg*_ than TiO_2_ is higher than that of TiO_2_ chiral film possibly due to stronger diffuse scattering effect diminishing the chiral response in the TiO_2_ chiral film, which is inferred from weaker interference fringes in the transmittance spectrum than that of SnO_2_ chiral film as shown in Fig. 2a 22. In case of the hetero-chiral film, two distinctive peaks are observed at 602 and 547 nm which corresponds to the circular Bragg phenomenon from the TiO_2_ and the SnO_2_ chiral film, respectively. Small deviation of *λ*_*0*_^*Bragg*^ peaks from those of the single-layer films may be due to slightly deviated *p* and *n*_*avg*_ of each layer in the hetero-chiral film during the sequential GLAD. Interestingly, the magnitude of the chiroptical response of the hetero-chiral film is much higher than that of single-layer films, which may be attributed to the combined response^17^,^23^ from the TiO_2_ and the SnO_2_ individual layers constituting the hetero-chiral film as well as the interference effect occurring at the hetero-interfaces.

**Figure 3:**
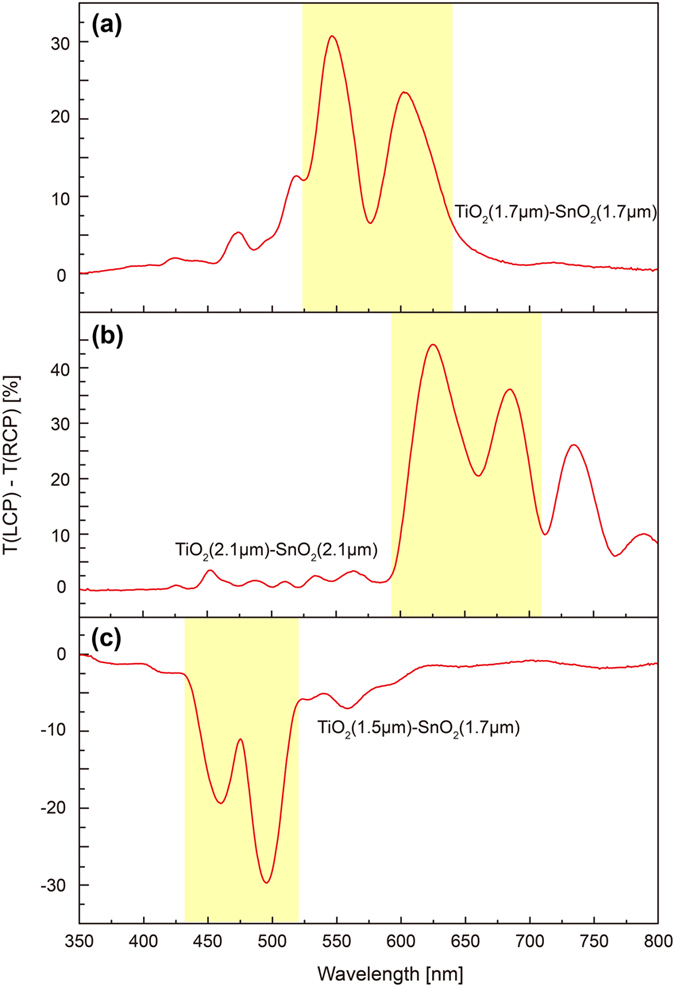
Difference in transmittance of circularly-polarized-light through various metal-oxides chiral films. The TiO_2_-SnO_2_ hetero-chiral films composed of five turns right handed single-layer chiral films with same pitches (*p*) of (**a**) 340 nm and (**b**) 420 nm. (**c**) The hetero-chiral films composed of five turns left handed single-layer chiral films with different pitches (300 and 340 nm for TiO_2_ and SnO_2_, respectively). Shaded region represents the wavelength range of the chiroptical response.

Note that a suitable combination of the circular Bragg phenomenon from selected materials constituting a hetero-chiral film results in not only strong chiroptical response but also excellent selectivity in the central Bragg wavelength and broad bandwidth near the wavelength of interest. In order to demonstrate such versatile advantages of hetero-chiral films, TiO_2_-SnO_2_ hetero-chiral films with different *p* and handedness were fabricated. Figure 3a–c show the difference in the transmittance of LCP and RCP light through the hetero-chiral films. Figure 3a shows the chiroptical response of the hetero-chiral film consisting of five-turns-RH helices of TiO_2_ and SnO_2_ thin films having same *p* (340 nm) (SEM image shown in Fig. 1c). Shaded region represents the wavelength range of the chiroptical response. According to Equation (1), the circular Bragg regime can be shifted toward a longer (shorter) wavelength by increasing (decreasing) *p* of the chiral film. Figure 3b shows the difference in the transmittance of LCP and RCP light through the RH hetero-chiral film consisting of five-turns-RH helices of TiO_2_ and SnO_2_ with longer *p* of ~420 nm than that of the film shown in Fig. 1c, resulting in red-shifted two circular Bragg peaks observed at 695 and 624 nm. The chiroptical response of the left-handed (LH) hetero-chiral film consisting of five-turns-LH TiO_2_ and SnO_2_ helices with slightly different *p* of 300 and 340 nm, respectively, is shown in Fig. 3c. Opposite sign of the curve is attributed to the handedness (LH) of the film reflecting LCP light while transmitting RCP light preferentially in the whole wavelength range. Cross-sectional SEM images of the hetero-chiral films and the chiral responses of single-layer films constituting the hetero-chiral films are provided in Supplementary information. Here it can be noticed from the spectrum (Fig. 3c) that the variation in *p* results in the two less-separated circular Bragg peaks (at 460 and 495 nm) comparing to the spectra of the hetero-chiral films of identical pitches (Fig. 3a,b) due to shorter *p* of the TiO_2_ LH-chiral film shifting the circular Bragg regime to a shorter wavelength. This indicates that not only the peak position but also the bandwidth of the circular Bragg regime can be tuned on demand by proper design of hetero-chiral films with appropriate combination of *n*_*avg*_ and *p*, which provides an elegant and simple way to realize circularly-polarized-light detectors with high wavelength-selectivity and broadband detection ranges necessary for various chiroptical applications.

### Circularly-polarized-light detector

**Figure 4:**
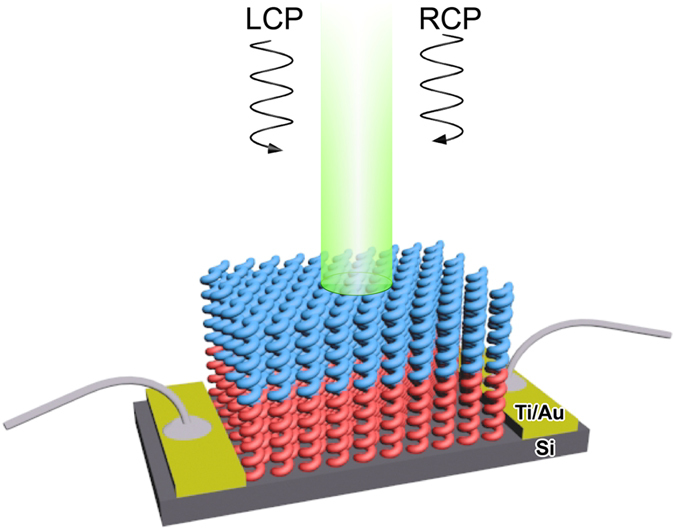
Schematic description of a circularly-polarized-light detector. Schematic description of a circularly-polarized-light detector based on metal-oxides hetero-chiral film under light illumination.

Figure 4 shows schematic description of a circularly-polarized-light detector based on a metal-oxide hetero-chiral film. The detector consists of a hetero-chiral film composed of two metal-oxides acting as an optical filter of circularly-polarized-light, undoped Si active layer, and Ti/Au electrodes (SEM images are provided in Supplementary information). It is worth to mention that such a device can be easily fabricated on a specific location of a microelectronic chip by conventional processes including photolithography for defining micro-dimensional area for sequential GLAD of chiral films and Ti/Au metallization for electrodes, followed by lift-off and wire bonding. Since circularly-polarized-light of opposite handedness to the chiral film transmits preferentially in the circular Bragg region, therefore, the light of opposite handedness induces more photocurrent in the active Si layer comparing to that of the same handedness. As the Si shows a high photo-sensitivity at long wavelengths (<~1100 nm), the hetero-chiral film with long *p* (420 nm) having chiroptical activity near long wavelengths (shown in Fig. 3b) is chosen for the fabrication of the circularly-polarized-light detector^24^.

**Figure 5:**
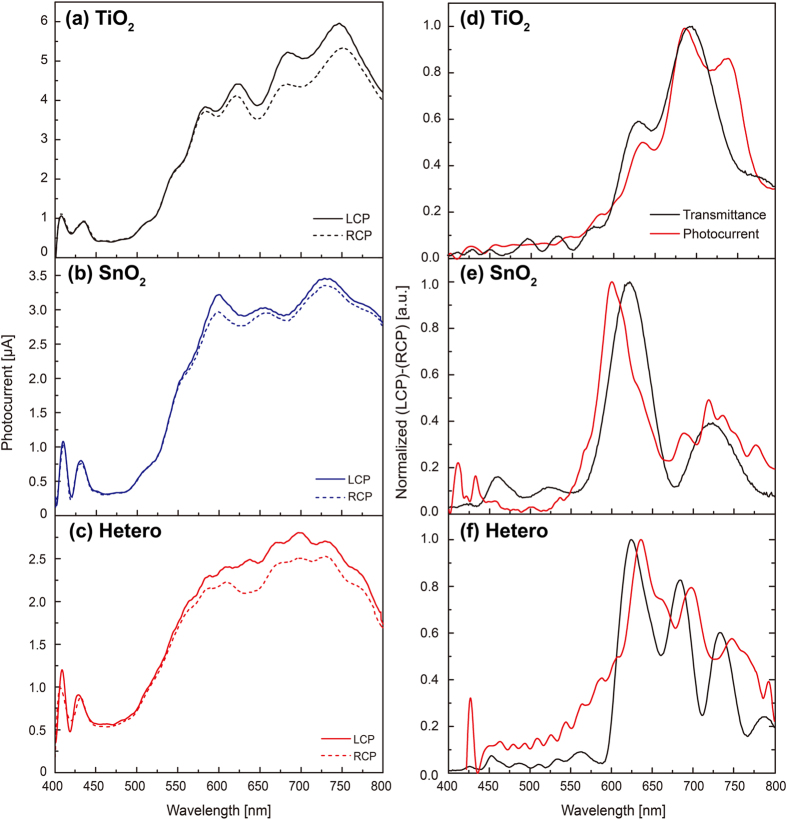
Photoresponse of photodetectors based on metal-oxides chiral films. (**a–c**) Photocurrent spectrums of the photodetectors based on chiral films described in Fig. 3b under circularly-polarized-lights at 0.1 V bias. (**d–f**) Normalized spectrum of the difference in photocurrents (black lines) under LCP and RCP incident light from the photodetectors based on the TiO_2_, the SnO_2_ and the hetero-chiral films, respectively. Normalized spectrum of the difference in transmittance (red lines) is shown for comparison.

Photocurrent spectra of the circularly-polarized-light detectors based on single-layer RH TiO_2_ and SnO_2_ chiral films, and RH TiO_2_-SnO_2_ hetero-chiral film under LCP and RCP light at 0.1 V bias are shown in Fig. 5a–c, respectively. Photocurrent under RCP light (dotted lines) is lower compared to that under LCP light (solid lines) near circular Bragg regime in accordance with chiroptical properties of the films. Lower photocurrent from the detector based on the hetero-chiral film than the single-layer chiral films is attributed to lower transmittance of the hetero-chiral film as shown in Fig. 2a. Figure 5d–f show the differences in both optical transmittance of the films and electrical photocurrent of the detectors for the incident LCP and RCP light. The TiO_2_ single-layer chiral film exhibits chiroptical response in longer wavelength than the SnO_2_ chiral film with same *p* (420 nm) due to higher *n*_*avg,TiO2*_ than *n*_*avg,SnO2*_. It can be noticed that the electrical photocurrent spectra of both detectors based on the TiO_2_ and the SnO_2_ single-layer chiral films correspond well to their chiroptical properties as shown in Fig. 5d,e. When those single-layer TiO_2_ and SnO_2_ chiral films are combined to form the TiO_2_-SnO_2_ hetero-chiral film by sequential GLAD, the chiroptical response occurs over much wider range and its magnitude becomes higher than the devices based on single-layer chiral films (Fig. 5f). Maximum percent change in photocurrent between LCP and RCP light is estimated to 15.8% from the hetero-chiral film based detector at 635 nm while 8% and 15.5% are estimated from the devices based on the SnO_2_ and the TiO_2_ single-layer chiral films at 646 and 683 nm, respectively (The spectra of the percentage change in photocurrents are provided in Supplementary information). Note that the difference in the percent change in photocurrent for LCP and RCP light between the single- and the hetero-chiral films is lower than that in the chiroptical responses from transmittance measurements. In addition, there are slight mismatches of the peak position between the transmittance and the photocurrent data shown in Fig. 5d–f. We believe that such deviations are attributed to the use of different substrates in each measurement, i.e., the glass substrate used for transmittance measurements while the Si substrate used for photocurrent measurements. The different refractive indices of the substrates cause the difference reflection at the interface between the chiral film and the substrate - higher reflection on the Si substrate occurs due to higher refractive index than the glass substrate. In addition, difference in surface properties may cause a slight difference in film morphology, resulting in such deviations.

The external quantum efficiency, defined as the ratio of the number of electrons flowing through the device to the number of photons incident upon it, of the hetero-chiral detector is estimated to be 30% even at low applied bias of ~0.1 V, much higher than previously reported value^10^. Consequently, a highly efficient circularly-polarized-light detector with excellent selectivity in an intended wavelength range is successfully demonstrated by combining geometrically-controlled hetero-chiral film as an effective chiroptical filter and the Si active layer having excellent carrier transport properties in an elegant and simple microelectronics-compatible way. For further development of the circularly-polarized-light detector integrated in the photonic circuit, it would be beneficial to predict and optimize the chiral films based on numerical calculations^12^,^13^,^16^,^25^.

## Methods

### Chiral film fabrication and characterization

All helical nanostructures in this work were fabricated by GLAD technique using an electron-beam evaporator. In GLAD method, by tilting the substrate with respect to the incident vapor flux, nuclei formed on the substrate at the initial stage of the deposition can provide self-shadow region to the subsequent vapor flux. A continuous supply of vapor flux makes these nuclei to grow in the direction of vapor flux, forming the slanted nanorods. The geometrical shape can be manipulated from inclined rod to vertical helix by using substrate rotation at a controlled speed. The *p* of the helical nanostructures can be precisely controlled by the substrate rotating speed relative to the deposition rate while the *n*_*avg*_ of the film can be controlled by choosing different refractive index evaporation material or the substrate-tilt angle which changes the porosity of the film^15^. In this work, the substrate-tilt angle was selected to be 65°, since it had shown maximum circular Bragg responses as a result of the competition between increased anisotropic nature and decreased refractive index of the film as increase the vapor incident angle^26^. Substrate rotating speed was set to 1 rpm while deposition rates of the TiO_2_ and SnO_2_ were optimized and kept constant to be 3.2 and 1.8 Å/s, respectively, in order to produce intended *p* (340 nm) of helical nanostructures. Only deposition rates were varied to achieve the helical films with different *p*. For example, deposition rates of 3.9 and 2.3 Å/s were chosen to produce the TiO_2_ and the SnO_2_ helical films with *p* of 420 nm, respectively. The refractive indices of the helical films with single-layer films were measured by using an ellipsometry. The surface morphology and elemental analysis were performed using field emission scanning electron microscopy (FE-SEM, Philips XL30s FEG). Optical characterization was done by measuring the transmittance of the chiral films on microscopic slide glasses using a spectrometer (Perkin-Elmer 1700). The circularly-polarized-light (RCP and LCP) were generated by inserting a linear polarizer and an achromatic quarter-wave plate oriented at ±45° with respect to the linear polarizer axis in the beam path of the spectrometer^15^,^18^,^27^.

### Device fabrication and characterization

The circularly-polarized-light detector based on chiral metal-oxides films were fabricated on an undoped Si. The active region (25 μm in width, 200 μm in length) where the chiral films were deposited was patterned by conventional photolithography followed by GLAD of metal-oxides and lift-off process. After formation of the chiral films on the active region, the samples were annealed at 500 °C for 2 hours in a furnace in order to enhance the crystallinity of the nano helices. Then, Ti (5 nm)/Au (50 nm) electrodes were deposited after standard photolithographic pattering, followed by lift-off process. Spectral photocurrent measurements were carried out at wavelengths from 400 to 800 nm with a white-light super-continuum laser source coupled to a monochromator and passing through the linear polarizer and achromatic quarter-wave plate creating circularly-polarized-light. The active regions were fully covered by uniformly irradiating laser spot, and bias voltage of 0.1 V was applied between two electrodes during the measurements. In order to obtain high signal-to-noise ratio, the illumination source was chopped and the photocurrent signal was measured using a source meter coupled to a lock-in amplifier. External quantum efficiency was estimated from the photocurrent spectrum of the hetero-chiral film based photodetector at 700 nm where it exhibits the maximum photocurrent under incident optical power of 15.86 μW.

## Conclusion

In conclusion, we demonstrated a highly efficient circularly-polarized-light detector with excellent wavelength-selectivity in an intended wavelength range by using a simple microelectronics-compatible processes including sequential GLAD. The circularly-polarized-light detector consisting of the geometry-controlled TiO_2_-SnO_2_ hetero-chiral thin film and the Si active layer shows excellent chiroptical response with external quantum efficiency of ~30% and high helicity selectivity of ~15.8% in an intended wavelength range. Furthermore, we demonstrated the ability of manipulating both bandwidth and responsivity of the detector in whole visible wavelength range simply by adjusting the combination of the geometry and the materials constituting hetero-chiral thin film. We believe that the high efficiency, wavelength selectivity and compatibility with conventional microelectronics processes enabled by the proposed device can result in remarkable developments in highly integrated photonic platforms utilizing chiroptical responses.

## Additional Information

**How to cite this article**: Lee, S. H. *et al.* Highly photoresponsive and wavelength-selective circularly-polarized-light detector based on metal-oxides hetero-chiral thin film. *Sci. Rep.* **6**, 19580; doi: 10.1038/srep19580 (2016).

## Supplementary Material

Supplementary Information
