# Supplementary material for: Highly photoresponsive and wavelength-selective circularly-polarized-light detector based on metal-oxides hetero-chiral thin film
Source: Sci Rep. 2016 Jan 22;6:19580. doi: 10.1038/srep19580 (PMC4726136; doi:10.1038/srep19580)
Supplement: Supplementary Information [file srep19580-s1.doc]

**Supplementary Information**

**Highly photoresponsive and wavelength-selective circularly-polarized-light detector based on metal-oxides hetero-chiral thin film**

Seung Hee Lee,^1^ Dhruv Pratap Singh,^2^ Ji Ho Sung,^1^ Moon-Ho Jo,^1^Ki Chang Kwon,^3,4^ Soo Young Kim,^3^ Ho Won Jang,^4^ and Jong Kyu Kim^1^,*

^1^ Department of Materials Science and Engineering, Pohang University of Science and Technology (POSTECH), Pohang, Gyeongbuk, 790-784, Republic of Korea

^2^ Max Planck Institute for Intelligent Systems, Heisenbergstrasse 3, 70569 Stuttgart, Germany

^3^ School of Chemical Engineering and Materials Science, Chung-Ang University, Seoul, 156-756, Republic of Korea

^4^ Department of Materials Science and Engineering, Research Institute for Advanced Materials, Seoul National University, Seoul, 151-744, Republic of Korea

* Corresponding author: kimjk@postech.ac.kr

1. **The refractive index and the absorption coefficient of the chiral films**

**
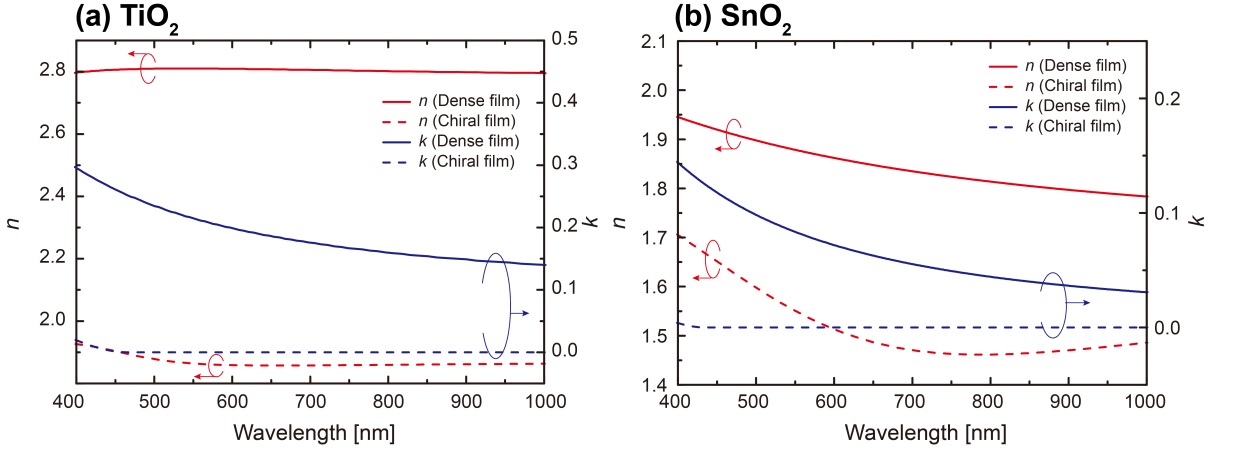
**

**Figure S1.** The refractive index (*n*, solid line) and the absorption coefficient (*k*, dotted line) of the **(a)** TiO_2_ and **(b)** SnO_2_ dense film (red) and chiral film (blue) fabricated with the substrate tilting angle of 65 ˚.

Figure S1 shows the measured refractive index (*n*) and the absorption coefficient (*k*) of the metal oxide dense films and chiral films, which were deposited with the substrate tilting angle of 0 ˚ and 65 ˚, respectively, by using ellipsometry. Thin film materials containing a high density of nanoscale pores can be considered as a composite of the depositing material and air; therefore, their refractive indices are different from those of the dense materials. Since the chiral films have been fabricated by GLAD with the substrate tilting angle of 65 ˚, the porosity increases due to the expanded self-shadowed region; therefore, the average refractive index of the thin film decreases. In result, the average refractive indices of the TiO_2_ and the SnO_2_ chiral film are lower than that of dense films.

1. **Geometric and chiroptical properties of various chiral films**


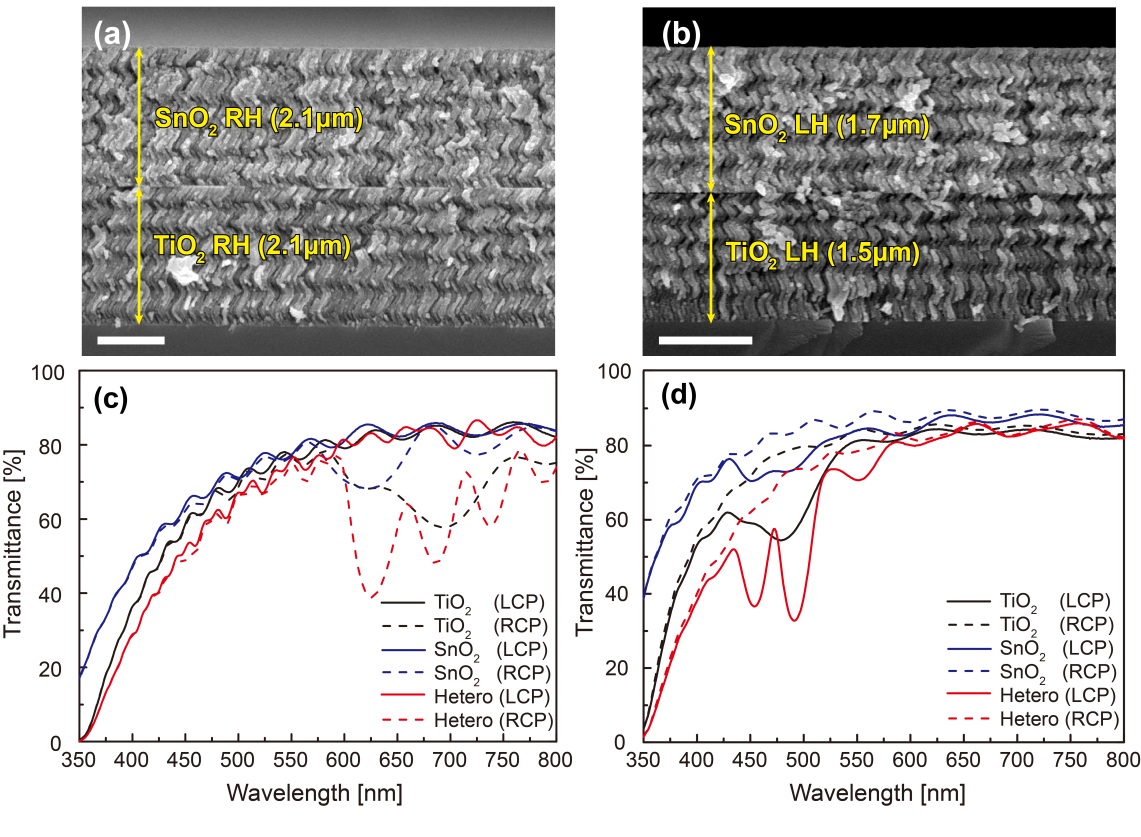


Figure S2. Cross-sectional SEM images of TiO_2_-SnO_2_ hetero-chiral films composed of five turns right handed single-layer chiral films with (a) same pitches (*p*) of 420 nm and (b) different *p* of 300 and 340nm for TiO_2_ and SnO_2_, respectively. (c-d) Respective transmittance spectra of RCP and LCP light through the chiral films as a function of the wavelength of incident light.

Figure S2 shows typical SEM images of the TiO_2_-SnO_2_ hetero-chiral film having different geometries and their chiroptical properties measured by transmittance under circularly-polarized light illumination. The difference in transmitted LCP and RCP light through the chiral film with long pitch (~420 nm) shown in Fig. 3b is acquired from the sequentially deposited five turns 2.1 μm thick-SnO_2_ and TiO_2_ RH chiral films (Figure S2a). RCP is reflected by the chiral films at wavelength in red range (624 and 695 nm) while LCP is transmitted regardless of wavelength as shown in Fig. 2Sc. On the other hand, a hetero-helical film composed of different materials and pitches (300 and 340 nm) is shown in Fig. S2b which corresponds to Fig. 3c. The LH hetero-chiral film reflects LCP at 460 and 495 nm exhibiting narrow distance between two circular Bragg peaks attributed to shorter pitch of TiO_2_ LH-chiral film shifting the peak to shorter wavelength according to Equation 1. Controllability of chiroptical properties by designing the hetero-chiral film with diverse materials and pitches can make the highly photoresponsive and wavelength-selective circular polarized light detector. Figures S3a~c show difference in transmittance of circularly-polarized light through the chiral films which is estimated from the transmittance spectrum as shown in Figs. 2a, S2c and S2d, respectively.


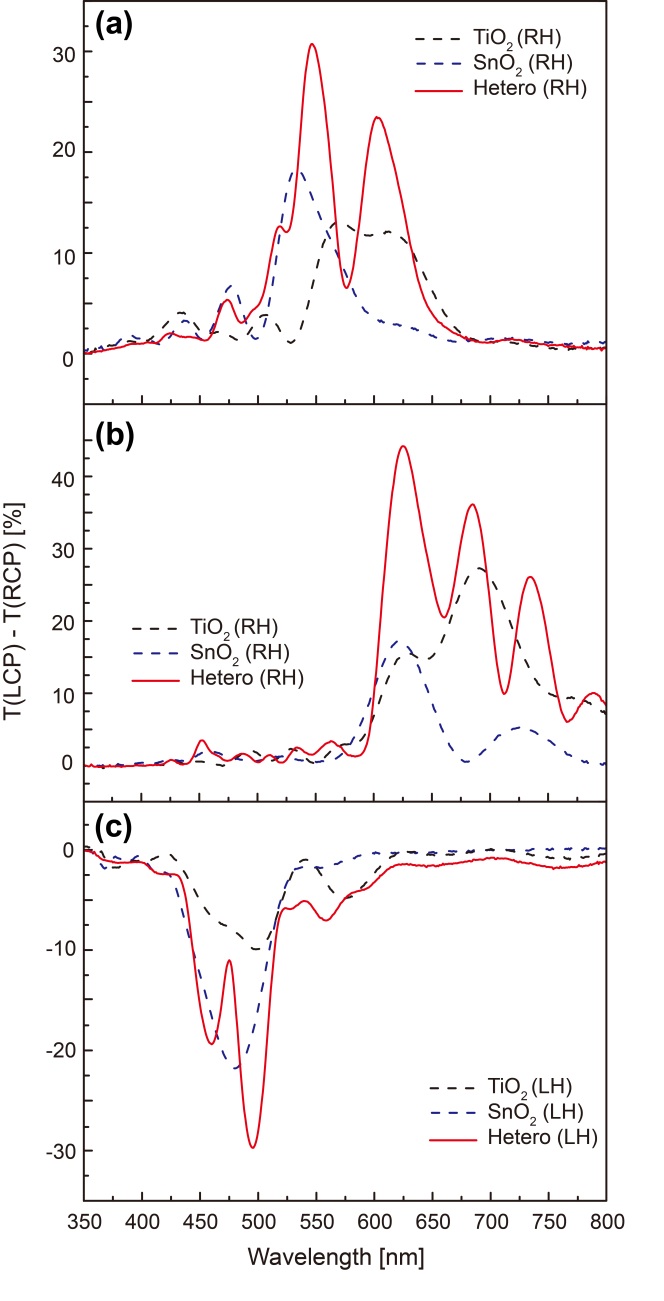


Figure S3. Difference in transmittance of LCP and RCP lights through TiO_2_ and SnO_2_ chiral films with the (a) same pitches of 340 nm, (b) 420 nm and (c) different pitches of 300 nm and 420 nm, respectively.

1. **SEM images of circularly polarized light detector based on metal-oxides hetero-chiral film**


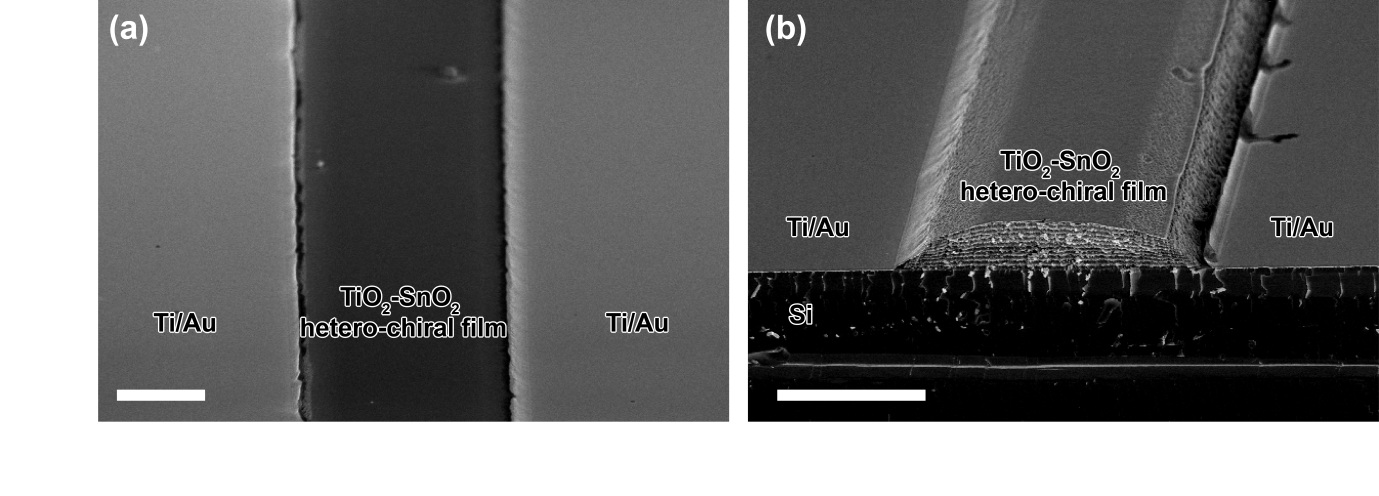


Figure S4. (a) Top-view and (b) bird’s-eye-view SEM images of a circularly polarized light detector based on TiO_2_-SnO_2_ hetero-chiral film. All scale bar indicates 10 μm.

Figure S4 shows typical SEM images of a circularly polarized light detector composed of TiO_2_-SnO­_2_ hetero-chiral film on Si active layer. The hetero-chiral films and a pair of Ti/Au electrodes were patterned by using photolithography processes to define photo-active region with width of 20μm. Not abrupt but gradual edge profile of the hetero-chiral film as shown in Fig. S4 is attributed to the shadow region formed by 3μm-thick photoresist during the GLAD process. Irradiated circularly polarized monochromatic laser beam within the active region can be selectively transmitted through the hetero-chiral film, and then reaches to active Si layer generating photocurrents.

1. **The spectrum of the percentage change in photocurrents**

**
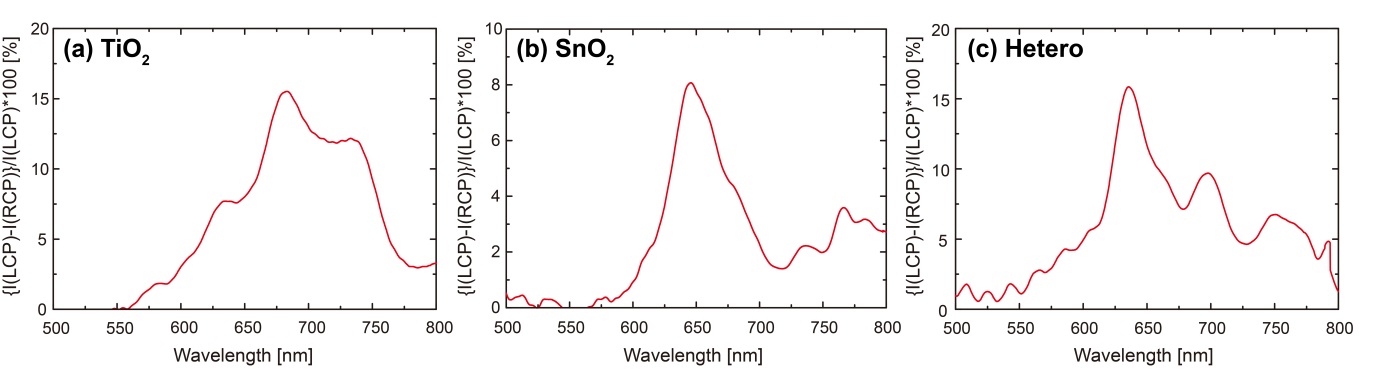
**

Figure S5. Spectrum of the percentage change in photocurrents estimated from Figs. 5a~c.

Figures S5a~c show the spectra of the percentage change in photocurrents under LCP and RCP incident light from the photodetectors based on the TiO_2_, the SnO_2_, and the hetero-chiral films, respectively. It can be noticed that the maximum percentage change in photocurrent for LCP and RCP light from the hetero-chiral film based detector (15.8 % at 635 nm) is higher than that from the devices based on the SnO_2_ (8 % at 646 nm) and the TiO_2_ (15.5 % at 683 nm) single-layer chiral films.
